# Patients’ pathways to cancer care in Tanzania: documenting and addressing social inequalities in reaching a cancer diagnosis

**DOI:** 10.1186/s12913-021-07438-5

**Published:** 2022-02-12

**Authors:** Fortunata Songora Makene, Richard Ngilangwa, Cristina Santos, Charlotte Cross, Twalib Ngoma, Phares G. M. Mujinja, Marc Wuyts, Maureen Mackintosh

**Affiliations:** 1 Economic and Social Research Foundation, 51 Uporoto Street, Ursino Estate, P.O Box 31226, Dar es Salaam, Tanzania; 2 Faculty of Arts and Social Sciences, The Open University, Walton Hall, Milton Keynes, MK7 6AA UK; 3 Department of Behavioural Sciences, Muhimbili University of Heath and Allied Sciences, United Nations Rd, Dar es Salaam, Tanzania; 4 International Institute of Social Studies, Erasmus University Rotterdam, Kortenaerkade 12, The Hague, 2518 AX The Netherlands

**Keywords:** Cancer, Patients’ pathways, Diagnosis, Access, Inequality, Delay, Costs, Disadvantage

## Abstract

**Background:**

This article investigates the extent and sources of late diagnosis of cancer in Tanzania, demonstrating how delayed diagnosis was patterned by inequities rooted in patients’ socio-economic background and by health system responses. It provides evidence to guide equity-focused policies to accelerate cancer diagnosis.

**Methods:**

Tanzanian cancer patients (62) were interviewed in 2019. Using a structured questionnaire, respondents were encouraged to recount their pathways from first symptoms to diagnosis, treatment, and in some cases check-ups as survivors. Patients described their recalled sequence of events and actions, including dates, experiences and expenditures at each event. Socio-demographic data were also collected, alongside patients’ perspectives on their experience. Analysis employed descriptive statistics and qualitative thematic analysis.

**Results:**

Median delay, between first symptoms that were later identified as indicating cancer and a cancer diagnosis, was almost 1 year (358 days). Delays were strongly patterned by socio-economic disadvantage: those with low education, low income and non-professional occupations experienced longer delays before diagnosis. Health system experiences contributed to these socially inequitable delays. Many patients had moved around the health system extensively, mainly through self-referral as symptoms worsened. This “churning” required out-of-pocket payments that imposed a severely regressive burden on these largely low-income patients. Causes of delay identified in patients’ narratives included slow recognition of symptoms by facilities, delays in diagnostic testing, delays while raising funds, and recourse to traditional healing often in response to health system barriers. Patients with higher incomes and holding health insurance that facilitated access to the private sector had moved more rapidly to diagnosis at lower out-of-pocket cost.

**Conclusions:**

Late diagnosis is a root cause, in Tanzania as in many low- and middle-income countries, of cancer treatment starting at advanced stages, undermining treatment efficacy and survival rates. While Tanzania’s policy of free public sector cancer treatment has made it accessible to patients on low incomes and without insurance, reaching a diagnosis is shown to have been for these respondents slower and more expensive the greater their socio-economic disadvantage. Policy implications are drawn for moving towards greater social justice in access to cancer care.

**Supplementary Information:**

The online version contains supplementary material available at 10.1186/s12913-021-07438-5.

## Background

This article analyses structured narratives by 62 Tanzanian cancer patients interviewed in 2019. The analysis focuses on the patterning by socio-economic inequality of the time elapsed, pathway patterns and costs of reaching a cancer diagnosis for these respondents. The article documents the socially unjust, regressive impact of delay on the costs experienced by these respondents in their search for diagnosis. It identifies implications for interventions that could make cancer diagnosis more rapid and accessible, and less socially inequitable, thus contributing towards more equitable Universal Health Coverage (UHC).

Access to cancer care in low-resource settings poses a major challenge for UHC. Tanzania was classified by the World Bank as a low-income country until 2020, now lower-middle income, with an estimated population of 58 million in 2019 [1]. Current health expenditure per head was just USD 37 in 2018^1^; of that, 24% was funded out-of-pocket, 1% from private insurance, 8% from social insurance (National Health Insurance Fund (NHIF)), 32% external funding, and 35% from domestic government expenditure [2].

The Tanzanian public health system is hierarchically structured. The primary level includes dispensaries and health centres and community outreach. District hospitals refer upwards as required to regional, then zonal and national including specialist hospitals. There are also government-supported and independent religious health facilities (17% of all registered facilities) and private facilities (18%) ([3]: 138).

Tanzania is experiencing rising cancer incidence and mortality [4] and is increasing the number of cancer treatment centres. Of 40,464 new cases in 2020, the leading cancers were cervical (25%), breast (10%) and prostate (9%) [5]. There is at present no systematic community-based screening for early-stage cancers; there is facility-based opportunistic screening capability for cervical cancer using Visual Inspection with Acetic Acid or Lugol’s Iodine [6]. In 2019, cancer treatment was available at one national specialist public hospital (Ocean Road Cancer Institute (ORCI)), in three zonal public referral hospitals, and in two private hospitals including Aga Khan hospital in the commercial capital, Dar es Salaam. Recognising that the high cost of chemotherapy and radiotherapy is a source of financial hardship and catastrophic spending for low-income populations [7], the Tanzanian government has exempted cancer patients from paying for treatment since introducing charging (cost sharing) into the public health sector in 1993. However, cancer patients continue to pay for other inpatient treatment, such as surgery and transfusions, and for diagnostic investigations and other medication.

In Tanzania as in many low- and middle-income countries (LMICs) most cancer patients are diagnosed late, reaching treatment only at late stages [8–10]. This sharply reduces treatment efficacy and survival rates [11–13] and multiplies the cost burden for patients and the health services. The gap between need and access to treatment remains wide [14] since many cancer sufferers in Tanzania are known to “never reach” treatment centres [15]. Accelerating diagnosis is thus a key Tanzanian policy objective [9], requiring an understanding of the sources of delay including inequity in barriers to care.

## Methods

### Data collection

This study used a structured qualitative research tool designed to explore barriers and facilitators experienced by cancer patients and survivors in Tanzania, along their pathways to and through cancer care, including interconnections between their experience and socio-economic status.

No sampling frame exists for selecting a representative sample of cancer sufferers and survivors in Tanzania. Respondents undergoing cancer treatment who were willing and able to be interviewed were recruited with medical staff assistance. At ORCI, the public specialist cancer referral hospital, two patient volunteers were requested from every other in-patient ward (40 patients), spreading the sample across the hospital. At Aga Khan Hospital, a faith-based private hospital, volunteers were requested both at the hospital and by contacting every fourth person on a data base of those under treatment; seven people agreed to an interview. To learn more of patients’ experiences at regional hospital level, nine interviewees with recent cancer diagnoses were recruited at Kitete (Tabora) or Tumbi (Pwani) regional hospitals (small numbers since these hospitals cannot currently offer treatment). Six cancer survivors were also recruited in Dar es Salaam with the assistance of survivors’ groups. The resultant convenience sample offered a wide spread of patients’ background and experience as the Results section confirms.

There was no selection of patients by type of cancer. The interview tool, attached as Supplementary material, drew on medical and health systems literature on clinical and care pathways [16, 17], but aimed particularly to contribute to the expanding exploratory qualitative literature on patients’ pathways to care in African contexts [18–21] especially patients’ experience including costs and other barriers to access [22, 23].

The interviews took the form of a structured conversation between interviewer and respondent, guided by the questionnaire. Respondents defined freely each event as they recalled it, from experiencing the symptoms later identified as indicating cancer (henceforth “first symptoms”) to diagnosis, treatment and beyond. For each event, the interviewees were asked: when or how long ago each occurred; what happened (e.g. symptoms worsened, returned for test results); where it occurred and what was done; and costs incurred including out-of-pocket (OOP) payments, costs covered by insurance or other sources, and travel costs.

Additional socio-demographic information included age, sex, schooling, current estimated monthly household income, place of residence and occupational status (current and before the onset of illness). Final questions covered use of alternative health treatments; insurance arrangements; participants’ social support and information networks; and open comments on their experience.

There was no audio recording. Interviewers recorded responses in writing, after a discussion if required to agree a response to each question; occasionally a lengthy or complex response was summarised. The interview tool was prepared in English and translated into and administered in Kiswahili. Responses were noted in Kiswahili, subsequently translated into English by each interviewer, and finally checked by supervisors.^2^ Participants shared these painful stories either privately or in a room with other patients or with carers who provided support. Participants did express a wish to tell their stories, and several said that they had felt listened to.

This research formed part of a larger collaborative project, *Innovation for Cancer Care in Africa.*^3^ Signed consent forms specified objectives and study content, privacy, data security, voluntary participation, and how to follow up.

### Data analysis

This article presents a structured qualitative analysis of elements of a substantial data set: 62 interviewees describing 906 events, plus additional data. The article uses both descriptive statistical analysis and thematic narrative analysis to analyse characteristics of patients’ pathways up to and including diagnosis, and to provide evidence of reasons for the delays observed.

The data set was coded numerically to identify socio-economic groups, to locate key milestones on each pathway (e.g, date of first experiencing symptoms, first health facility visit, event when cancer diagnosed), and to identify levels and sector of all facilities visited. Descriptive statistical analysis used Stata 14.

Descriptive statistical findings provide evidence of the variation in extent of delay before diagnosis, and the patterning of delays by socio-economic background and by other relevant quantifiable variables such as out-of-pocket spending and insurance arrangements. The empirical distributions of the variables in the descriptive statistical analysis are asymmetric and strongly skewed with the tail stretching towards larger values. The findings are therefore presented using order-based rather than mean-based summary measures. The tables list sample size (N) and three-number summaries: the lower quartile, median and upper quartile of each empirical distribution. This presentation allows inspection of degree of skewness, a relevant dimension of the data for exploring socio-economic inequality.

Thematic qualitative analysis was also conducted of patients’ narrative responses, to identify reasons given for delay and to explore how these were related to individuals’ socio-economic characteristics. Thematic coding used NVivo12, with the data uploaded in survey mode. A priori coding used the preidentified themes in the questionnaire, including symptoms, diagnostic tests, costs, travel. Additional coding identified themes relevant to explaining delay, including referral (and lack of referral), funding sources, reasons for moving between providers.

The presentation of results combines descriptive statistics to identify patterns in the data and narrative cases, summarising experiences of selected individual patients, chosen to illustrate identified themes contributing to explanation of delays and their patterning by dimensions of socio-economic inequality. This use of descriptive statistical analysis in association with explanatory narrative case material is an accepted approach to qualitative research and analysis in social science [24]. Descriptive statistics identify patterns and allow cross checking of typicality of emergent themes, thereby helping to establish that findings from thematic analysis are indeed characteristic of the whole set of individuals studied (“internal generalizability”) while making *no* inferences beyond the sample ([24]: 477–8).

## Results

Descriptive statistical analysis demonstrates that the most disadvantaged patients in our data set generally faced the longest delays before diagnosis. A combination of descriptive statistics and narrative case studies of patients’ experience identify key reasons for delay and their patterning by disadvantage. The results starkly foreground the socially unjust cumulative impact of these factors in delaying cancer diagnosis while imposing a severe financial burden on the most disadvantaged of our respondents.

### Intersections of disadvantage and delay

#### Indicators of relative disadvantage

Three indicators of relative deprivation and socio-economic inequality used in this analysis, education level, type of occupation and declared monthly household income, are closely related among our respondents. All those with post-secondary education declared professional occupations (two retired), while those with below-primary education were predominantly farmers (Table 1).

**Table 1:** Level of education and occupation before illness (number of respondents)

Occupational category	Education level			
	Below primary	Primary and secondary	Above secondary	All respondents
**Farmer**	12	15	0	27
**Business/ self employed/ casual work**	5	15	0	20
**Professional/ retired**	0	5	10	15
**All respondents**	17	35	10	62

Pearson chi2(4) = 40.986 Pr = 0.000

Source of all tables and figures: authors’ data

The non-farming business/ self-employed category is broad, from viable businesses through casual labourers; these data do not allow finer distinctions. Declared household income reflects both educational and occupational hierarchies. Farmers’ incomes and incomes of those with below-primary education were low and compressed (median TZS 100,000 and 150,000/month respectively (USD 43 and 65)). Most declaring higher incomes held post-secondary qualifications. These socio-economic indicators did not differ significantly between men and women in the data set.

The cancer patients interviewed in the public sector (in ORCI and two regional hospitals) were overwhelmingly from low income, low education and economically precarious backgrounds. As such, their levels of disadvantage reflect the Tanzanian population. Of 49 such patients (71% female), 90% had just primary education or below, including 11 with no formal education. Declared monthly household income of 51% of interviewees was below TZS 200,000 (USD 87),^4^ and most declared their occupation prior to illness as farmer (55%), self-employed business or casual work (18%), or not working; only three had had professional employment. For comparison, on the Tanzanian Mainland, average household consumption expenditure in 2017/18 was USD 181 ([25]: 8). Employment in agriculture was an estimated 63% of the total in 2018 [26], while the share of non-agricultural self-employment and casual work in 2014 was estimated at 20% [27]. Finally, patients interviewed at ORCI came from many regions. These data are consistent with a perception that ORCI treats cancer patients from across Tanzania’s social, economic and geographical spectra, not just from a privileged group.

Conversely, the private hospital patients and the Dar es Salaam-based cancer survivors (62% female) formed a generally less disadvantaged group. Of these 13 respondents, nine had secondary education or above; seven had degrees; and eight declared household incomes of TZS 500,000/month (USD 217) or above.

#### The association of disadvantage with delay

Respondents’ narratives documented a frequently lengthy search for care: median delay between first symptoms and cancer diagnosis was 358 days. Nearly half had waited over a year, and 28% more than 2 years for a diagnosis. The extent of this delay was strongly patterned by relative disadvantage.

Length of delay between first symptoms and diagnosis fell steeply and consistently across the distribution as education levels rose (Table 2). The median delay for the least educated category was nearly 2 years (Table 2).

**Table 2:** Cumulative time from first symptoms to diagnosis by education level (quartiles of distribution of cumulative days)

Education	N	p25	Median (p50)	p75
Below primary	15	274	670	1827
Primary/ secondary	33	154	381	762
Diploma/degree	10	25	128	276
**All respondents**	^**a**^**58**	**124**	**358**	**763**

^a^Date of diagnosis is unclear for four respondents

Those with professional occupations experienced the shortest delays across the distribution, while self-employed and casual workers had consistently experienced the longest delays (Table 3).

**Table 3:** Cumulative time from first symptoms to diagnosis by occupational category (quartiles of distribution of cumulative days)

Occupational category	N	p25	Median (p50)	p75
Farmer	26	124	387	762
Business/ self-employed/ casual	17	425	670	1493
Professional	15	25	135	276
**All respondents**	^**a**^**58**	**124**	**358**	**763**

^a^Date of diagnosis is unclear for four respondents

### Sources of delay and facilitators of diagnosis

What were the main reasons for delay, and why were they so strongly patterned by relative disadvantage in this data set? Patients’ narratives identify three key sources of delay: lack of diagnostic capacity and speed, including at regional hospital level; dominance of self-referral within the system as patients struggled to reach a diagnosis; and sharply regressive out-of-pocket costs imposing a search for funds and delaying diagnosis. Conversely insurance and private sector access had accelerated diagnosis for the more privileged.

#### Diagnostic delay and the failure of “by-passing”

Most of the delay before diagnosis occurred *after* patients first went to a health facility. At first symptoms, 66% of respondents said they went directly to a health facility. For the others, the median initial delay was 3 months. Much the longest delays after first going to a facility were again experienced by the least educated group.

Since public sector cancer diagnostic capability is currently concentrated at regional hospital level and above, it was expected that patients who went directly with first symptoms to a higher level facility (often called “bypassing” [28]) would achieve a more rapid diagnosis. Strikingly, this was *not* case. Patients who had started at the higher level in the public sector had taken longer, across most of the distribution, to reach a diagnosis than those who had started lower down in the public sector; half had waited 1.5 years or more (Table 4).

**Table 4:** Cumulative time from first visiting a health facility to diagnosis, by level of entry into the formal health system (quartiles of distribution of cumulative days)

Level of entry	N	p25	Median (p50)	p75
Primary	15	152	394	703
District hospital	19	88	290	725
Higher level public hospital	13	117	547	1506
Private sector facility	11	11	135	336
All respondents	^a^58	58	197	703

^a^Date of diagnosis is unclear for four respondents

A large majority (82%) of respondents went first to a public facility (Table 4), and there was *no* significant patterning of health system level of entry in the public sector by occupation or education: that is, those “by passing” were not more advantaged. Only starting in the private sector had sharply reduced the delay before diagnosis (Table 4). Just 11 patients had done so (Table 4), predominantly professionals with higher education.

Patients’ narratives show that a lack of diagnostic capacity, particularly at regional hospital level, and especially in pathology for processing biopsies and in imaging, exacerbated by staff shortages and slowness in recognising symptoms, were the main reasons why diagnosis was often slow even at higher levels and why “by-passing” had not been a route to rapid diagnosis for these patients. Case 1 exemplifies several causes of diagnostic delay.

##### Case 1^5^ diagnostic delay at regional level

This patient went directly to a regional hospital with first symptoms that could indicate cervical cancer (vaginal discharge, swelling, pain). Over six visits in 6 months, she was given urine tests, medication including antibiotics, and once an ultrasound. As symptoms worsened and wounds opened, she went first to a nearby health centre, then to a dispensary, and was given further medication. She returned to the regional hospital twice, 16 months and 21 months after the first visit, with similar outcomes. She then turned in despair to traditional healing for 6 months, and then for several months to prayer. She returned to the same regional hospital in severe pain three and a half years after the first symptoms. There, she was referred to a private clinic for a biopsy. The patient was then asked by the regional hospital to take the biopsy sample to the national hospital in Dar es Salaam for analysis. Five months later, the diagnostic results were received at the regional hospital. The patient was then referred to ORCI with cervical cancer. (Female patient, self-employed, primary education).

Conversely, where regional diagnostic capability did exist, patients had benefited through rapid diagnosis. For example, one patient (male, below-primary education, farmer) with severe leg symptoms had delayed 3 months before going to hospital. Once there however, the regional hospital took a biopsy, and diagnosed skin cancer within a month. The patient reported that the biopsy cost TZS 200,000 (USD 87).

#### Self-referred “churning” within the system: a source and response to delay

A second major source of stress and delay identified from patients’ narratives, also well-illustrated in Case 1, was the experience of moving repeatedly between facilities in search of a diagnosis. Just 5 respondents were diagnosed at their first facility (Table 5 Row 2). The more moves in search of help, the longer the delays across the distribution before diagnosis (Table 5).^6^

**Table 5:** Cumulative time to diagnosis by number of moves between health facilities (quartiles of distribution of cumulative days)

Number of moves between facilities	N	p25	Median (p50)	p75
No move	5	25	124	454
1 move	11	11	117	276
2 moves	20	132	229	777
3 moves	10	274	686	763
4–8 moves	12	588	1010	3471
All respondents	^a^58	124	358	763

^a^Date of diagnosis is unclear for four respondents

Like the Case 1 patient, 40% of respondents had moved “downward” in the facility hierarchy while seeking a diagnosis, including over half of those initially “by-passing” to regional or zonal hospitals. Those starting at higher levels had moved around just as much as those who started lower down.

These moves, which we call “churning” within the system, were undertaken largely without formal referral. Among patients not diagnosed at the facility they first visited, 55% had never been formally referred before diagnosis, yet had moved a median of twice, while the others had at least one referral during their search for diagnosis yet had moved a median of three times. Most moves were thus self-referred: for example, of 16^7^ people who started at dispensary or health centre level, just two had been referred upwards; all others reported self-referring to a hospital.

Churning had most seriously affected those with the lowest education: 53% of those with below-primary education had moved more than twice, as had 41% of those with primary or secondary education. Conversely, only 1 person with higher education (10%) and 2 professionals (13%) had moved more than twice.

Patients’ experience was therefore predominantly of self-referred moves in search of help in the face of worsening symptoms rather than patients following pathways structured by the health system, as further illustrated in Case 2.

##### Case 2 self-referred “churning” and associated delay

This patient went directly to a regional hospital with low back pain. Over several years she went back three times and was given pain medication and her back X-rayed. As stomach pain also developed, she then went successively to a faith-based hospital, a district hospital and a dispensary. Then, struggling with self-described “unstoppable” stomach and back pain, she went back to the district hospital. Imaging and pain management still did not produce a diagnosis. By chance she then went to a free screening session at a church and was told this was cervical cancer. She returned to the same regional hospital, where a biopsy was taken and she was told by the doctor to come back after 3 months for the result. On her return the diagnosis was confirmed. She estimated that by then, she and her family had spent TZS 540,000 (USD 235) on her search. (Female patient, completed primary school, not working (for gain)).

Conversely, some patients’ narratives confirm that ability to recognise symptoms at lower levels and formal referral at an early stage could speed up diagnosis, particularly where the referring facility expressed suspicion of cancer. For example, one patient went to a district hospital with stomach pains. The hospital examined her cervix and sent her straight to ORCI. Within a month she was diagnosed and started radiation treatment. (Female, farmer, did not complete primary education). Similarly, a regional hospital with better capability had responded rapidly to a patient referred from a health centre with bleeding, discharge and pain. Although tests took a month to return, the hospital diagnosed uterine cancer within 3 months of the patient first presenting symptoms at a dispensary. (Female patient, farmer, completed primary school).

#### Use of informal providers and health system delays

In addition to moves between health facilities, respondents had also visited drug shops, pharmacies and alternative forms of healing, adding to churning and resultant delays. Those with lower education were more likely to move between health facility and informal providers, while 90% of those with higher education went only to health facilities.

Only four people went to a herbalist or traditional healer *before* going to a health facility. Seventeen others visited them as part of their search for diagnosis. Case 1 illustrated recourse to traditional healing after many failed attempts to find relief from symptoms within the formal system. Others had followed advice from family and friends. These visits were sometimes influenced by experiences of formal health care; they could thus be both result and cause of delay. Case 3 gives a further example.

##### Case 3 combining health facility and traditional healer visits

A patient with vaginal symptoms was advised by her children to go to a dispensary. There she was given antibiotics and pain killers. Six months later the symptoms had worsened, and, advised by neighbours, she went to traditional healers. Five months later with worsening symptoms she recounted that she decided to stop using local herbs while her daughters were searching for money so that they could take her to hospital. She then went to a faith-based hospital where cervical cancer was diagnosed. (Female patient, primary education, farmer).

#### Regressive payment burdens as a source of delay

Patients had faced highly regressive out-of-pocket (OOP) costs, falling most heavily on those with lowest incomes, during their search for diagnosis. The impact on delay was cumulative and fell most heavily on the most disadvantaged.

The cumulative total of OOP payments ﻿rose shockingly as “churning” increased (Table 6).^8^ Those who made more than two moves faced strikingly higher payments (Table 6).

**Table 6:** Cumulative OOP payments to providers (formal and informal) from first symptoms to diagnosis, by the number of times a patient moved between providers (quartiles of distribution of cumulative payments; Tanzanian shillings; USD in brackets for median payments)

Number of moves between providers	N	p25	Median (p50) (USD)	p75
No move or 1 move	9	0	0 (0)	200,000
2 moves	10	0	34,500 (15)	145,000
3 or 4 moves	12	155,000	557,000 (242)	786,250
5–10 moves	6	298,000	436,500 (190)	1,508,000
All respondents	^a^37	2500	170,000 (74)	700,000

^a^number of patients able to recall 70% or more of payments per recorded event

The burden of these OOP payments to health providers, including payments to traditional healers, pharmacies and drug shops, was sharply and consistently regressive, imposing the heaviest burden on the most disadvantaged (Table 7). The lowest educated had borne the heaviest *absolute* payment burden; for those with post-secondary education, the median payment was zero. Our sample necessarily includes only those sufferers who had managed to raise the required money: it is likely others will have dropped out of the search for care.

**Table 7:** Cumulative OOP payments to providers (formal and informal) from first symptoms to diagnosis, by level of education (quartiles of distribution of cumulative payments; Tanzanian shillings; USD in brackets for median payments)

Level of education	N	p25	Median (p50)	p75
Below primary	10	170,000	547,500 (238)	790,000
Primary/ secondary	19	10,000	220,000 (96)	540,000
Diploma / degree	8	0	0 (0)	73,750
**All respondents**	^a^37	2500	170,000 (74)	700,000

^a^number of patients able to recall 70% or more of payments per recorded event

Since declared household income rose with education, these regressive payments created a very severe financial burden for the most disadvantaged respondents (Table 8). For over a quarter of the patients in the lowest income band, recorded cumulative OOP payments up to diagnosis exceeded annual declared household income.

**Table 8:** Household burden of cumulative OOP payments to providers (formal and informal) from first symptoms to diagnosis (quartiles of cumulative payments divided by *annual* household income)

Bands of declared household monthly income	N	p25	Median (p50)	p75
Up to 150,000	12	0.068	0.366	1.325
160,000 - 390,000	13	0.002	0.065	0.093
400,000 upwards	12	0	0.004	0.157
**All respondents**	^a^37	0.001	0.072	0.313

^a^number of patients able to recall 70% or more of payments per recorded event

Transport costs formed an additional burden exacerbated by churning and distance to higher level hospitals. Median cumulative transport spending up to diagnosis was TZS 83,000 (USD 36),^9^ rising from TZS 16,000 (USD 7) for those diagnosed at their first facility to TZS 92,000 (USD 40) for the majority who moved more than once.

Respondents’ narratives indicated that costs of diagnostic tests, some of which required surgery, were a leading source of stress and delay. Table 9 summarises reported payments for imaging (CT scans, ultrasound, MRI), biopsies and surgery, all expensive in relation to reported household incomes (Table 8).

**Table 9:** OOP payments by type of service purchased at each event from all providers (formal and informal) from first symptoms to diagnosis (quartiles of payments per service; Tanzanian shillings; USD in brackets for median payments)

Service paid for	^**a**^N	p25	Median (p50)	p75
Admission plus other services	25	30,000	115,000 (50)	600,000
Imaging, biopsy	54	33,000	80,000 (35)	200,000
Tests, medication	80	5000	14,000 (6)	30,000
Consultation, referral	19	5000	15,000 (7)	50,000
Alternative healing	28	20,000	30,000 (13)	80,000

^a^N in this table = number of events at which payments for the service were recorded

Cases 4 and 5 below illustrate the reported burden of finding money to pay for biopsies and other costs, and the resultant delays.

##### Case 4: the financial burden of biopsies and other costs

This patient went twice to a dispensary with swelling on the head and was given injections and pain killers. Eight months later with severe headache and more swelling he went twice to a district hospital where he was checked for typhoid and TB and given medication. Four months later with severely worsening symptoms, he underwent a biopsy and blood tests at a faith-based hospital and was told to come back after 2 months. Given a cancer diagnosis, he was referred to a zonal hospital where he received stronger pain medication and a further biopsy confirmed cancer. The two biopsies cost TZS 150,000 and TZS 400,000 respectively (total USD 240), and the patient calculated that up to that point the family had spent TZS 640,000 on medical care and TZS 175,000 on transport to facilities (total, USD 344), funded by selling two cows. This patient’s declared monthly household income was just TZS 50,000. (Male patient, primary education, farmer).

##### Case 5: delays arising from costs of tests

This patient went to a district hospital with a breast lump and was referred to a zonal hospital. Over four months and in increasing pain, two consecutive biopsies showed no cancer. She then went to three different traditional healers over more than a year. With multiplying symptoms, she then went to a faith-based hospital who referred her back to the zonal hospital. There she was given ultrasound and X-ray examinations, but she lacked the money to pay for a further biopsy. Five months later, advised, she said, by a neighbour whose husband is a doctor, she returned to the zonal hospital where she paid for a biopsy and new ultrasound and X-ray examinations. Two months later she received a cancer diagnosis. The patient could not remember every payment during this lengthy “churning” but calculated that investigations at the zonal hospital totalled TZS 305,000, while the traditional healing had cost TZS 410,000 and transport at least TZS 97,200: total TZS 812,200 (USD 353). This patient had declared monthly household income of just TZS 60,000 (USD 26). (Female patient, completed secondary, self-employed business).

There were many such examples of the pain and stress from biopsies delayed because patients could not pay to undergo them, nor to take them to a hospital with pathology services. These payments both delayed diagnosis and undermined patients’ household finances – permanently if assets were sold.

#### The private sector and insurance access: facilitation and inequality

While expensive to access, the private sector had played a facilitating role in accelerating diagnosis for those able to pay or having access to insurance. Most (60%) of all respondents held no insurance. The others held two different types: “strong” insurance, within which we include membership of the National Health Insurance Fund (NHIF)^10^ and/or private insurance funds such as AAR (31%); or “weak” insurance such as membership of a Community Health Fund (CIF) with limited coverage or exemptions from certain facilities’ payments (10%).^11^ Strong insurance was held by 73% of professionals and 90% of those with higher education, but just 17% of interviewees with other occupations and 19% with lower educational levels. Strong insurance very sharply reduced the level of OOP payments before diagnosis, reducing the median payment to zero (Table 10). No-one without strong insurance escaped without any OOP payments.

**Table 10:** Cumulative OOP payments to providers (formal and informal) from first symptoms to diagnosis, by insurance status (quartiles of distribution of cumulative payments; Tanzanian shillings; USD in brackets for median payments)

Insurance status	N^**a**^	p25	Median (p50) (USD)	p75
None	19	140,000	540,000 (235)	1,251,300
Weak	4	117,000	281,500 (122)	516,500
Strong	14	0	0 (0)	35,000
**All respondents**	^a^37	2500	170,000 (74)	700,000

^a^N: number of patients able to recall 70% or more of payments per recorded event

Strong insurance also gave access to the private sector: 90% of those whose first visit was to a private health facility held strong insurance, and those respondents experienced much less churning and delay. The private sector had also facilitated cancer diagnosis for six patients who started their pathways in the public sector, but at a cost which could add to delay and financial burden.

##### Cases 6 and 7: strong insurance facilitating rapid private sector diagnosis

A patient holding private insurance experienced severe breast pain. Despite reluctance, she was persuaded by work colleagues to go directly to the large private hospital, Aga Khan. There a mammogram and biopsy provided a diagnosis of breast cancer in 2 weeks. All charges were paid by insurance. (Female professional with higher education).

A second patient holding both NHIF and private insurance experienced severe stomach pain and vomiting and went to a private facility. There a CT scan identified a liver problem. She was advised to go to a second private hospital for an MRI and was then diagnosed with liver cancer in less than a month at Aga Khan hospital. Insurance paid all these costs. (Female professional with higher education).

### Inequality-reinforcing factors in access to cancer diagnosis

Several key socio-economic factors were thus mutually reinforcing in reducing delay before diagnosis: better education, professional occupation and strong insurance coverage. These characteristics of interviewees were associated with intermediate factors within the health system that reduced delays: private sector starts, fewer moves between providers in search of care, and a lower cost burden.

The combined regressive impact for our respondents of these cumulative influences is starkly demonstrated in Table 11. Respondents are divided into 12 “more privileged” individuals with at least two of the following characteristics: professional occupation, post-secondary education and strong insurance; 33 “more disadvantaged” people with primary or secondary education; and 17 individuals with below-primary or no formal education (Table 11).

**Table 11:** Impact of relative privilege

Item and measure	More privileged	More disadvantaged	Very low education
Cumulative days to diagnosis (median)	137	394	670
Cumulative days from first health facility visit to diagnosis (median)	134	200	599
Number of moves between providers^a^ before diagnosis (median)	1.5	3	3
First facility visited was in private sector (%)	75%	6%	0
Cumulative OOP payments up to diagnosis (TZS) (median)	0	225,000	700,000

^a^All providers including health facilities, drug shops, pharmacies and traditional healers

The “more privileged” group experienced the shortest delays before diagnosis and the lowest levels of “churning”. They had generally started their pathways in the private sector, and the median out-of-pocket spending of this group before diagnosis was zero. Conversely, those with very low education, though they had *not* started at lower level facilities than others starting in the public sector, had consistently the longest waits, and had paid the largest sums in OOP spending to reach a cancer diagnosis.

## Discussion

Our core finding, summarised in Table 11, is the socially unjust patterning of these cancer patients’ experiences by education, occupation and income. The greater the social and economic disadvantage, the longer the delay, the greater the stress of self-referral, and the higher the absolute financial burden. The findings also illustrate the mutually reinforcing effects of socio-economic disadvantage and public health system capability weaknesses. There are strong, inequality-reinforcing interlinkages between what are sometimes called [19] patient-mediated (e.g. socioeconomic status) versus health system-mediated (e.g. referral problems) delays to cancer care access.

The strength of these findings is their rootedness in patients’ experience: there is a dearth of evidence in the literature on cancer patients’ perspectives from lower income countries. In analysing patients’ experiences up to diagnosis, this article extends the limited existing literature on the cost and barriers to accessing cancer care in Tanzania [14, 29–31]. While *no* statistical claim is made for generalisability to the Tanzanian population, the consistency of the findings within the data set, and their external consistency with other evidence including OOP payments as barriers to care and estimated drop-out from cancer care, support their relevance for policies aiming to move towards UHC including greater social justice in access to cancer care.

Key policy implications, largely consistent with existing literature, include the following. Diagnostic capacity in Tanzania is heavily concentrated in 7 zonal and national hospitals, located in 5 major cities in Tanzania. Only four consultant hospitals, in addition to some private and faith-based facilities, have a pathology laboratory ([12]: 8), meaning that regional hospitals frequently do not have the capacity to analyse biopsies. As some of our respondents experienced, such challenges potentially delay the diagnosis and referral of potential cancer patients [29, 32]. Furthermore, magnetic resonance imaging and computerized tomography scans are often not available at regional hospital level, meaning patients must travel to zonal or national facilities [33–35]. Strengthening the diagnostic skills and capabilities of the regional hospitals, including capability to undertake biopsies and laboratory pathology, implies substantial investment but would improve the referral role of facilities that are much more geographically accessible to patients: this could be a focus for external funding support.

Our findings on the high costs of reaching a diagnosis complement qualitative evidence from elsewhere in the region on OOP costs of accessing cancer care [36–39]. We add sharper evidence on the highly regressive impact of the OOP spending, including examples of impoverishment through sale of assets, and the impact of delays on costs, self-referral and travel in search of care. Our findings support the importance of policy towards diagnostics in UHC initiatives [40]. Policy changes that might reduce OOP cost and speed up diagnosis include subsidising diagnostic tests to reduce cost sharing and formalising public/private arrangements to enable greater access to testing infrastructure held in the private sector. Our findings confirm the benefits of insurance for speeding access to diagnosis through more rapid private sector response, and the impact of this on inequality of costs and speed of diagnosis. Financial reforms are required, consistent with the principles of UHC, that promote integration of financing schemes and improve financial support for the least well-off who lack insurance and struggle to meet OOP costs.

Cervical cancer screening is being extended systematically in Tanzania ([41]: 15); however, coverage remains limited and identified barriers to attendance include limited knowledge and local availability as well as anticipated cost of travel and the procedure itself [8, 42]. This is consistent with our finding that just one patient interviewed had been diagnosed (after facility visits) through screening. Our evidence on delayed investigation of symptoms strongly suggests that more systematic screening has scope to reduce sharply diagnosis times for some cancers. However, realising potential requires initial diagnostic screening, such as visual inspection for cervical cancer, to be more rapidly confirmed by biopsies when required.

Our finding that those with lowest education faced the longest delays is consistent with existing literature on Tanzania and other African countries that suggests limited awareness among the wider population of symptoms of cancer, alongside stigmatisation and fear of screening and treatment, contribute towards late diagnosis of cancer, prompting calls for wider public information [6, 36, 43–48]. However, patients interviewed had showed no “lack of personal initiative” ([8]:1) in seeking care. Many had taken family, friends’ and neighbours’ advice, and were very active in self-referral; use of traditional healers was often a response to health system delay.

Our evidence demonstrated however the limitations of formal referral as it is currently functioning for cancer patients, a weakness acknowledged within government policy ([49]: 10). While anthropological research has shown how oncologists and nurses may have to ‘improvise’ in the provision of cancer care in low income settings [50], this research illustrates the extent to which patients without strong insurance must also try to piece together care that is acceptable, affordable and available to them as they seek a diagnosis. This echoes a smaller qualitative study of cancer patients’ experiences in Tanzania in suggesting the potential for misdiagnosis and provision of inappropriate, but often costly, initial treatment to contribute to late diagnosis [31].

In summary, this article demonstrates the extent to which health-seeking behaviour by our respondents was, as in other studies, shaped by economic hardship [21] and by perceived failings and limitations of the formal healthcare system [48, 51]. However, just as aspects of disadvantage were shown to be mutually reinforcing, so partial solutions can potentially create a virtuous circle of interactions between better facility capabilities, lower charges, widening insurance, better information, strengthened referral pathways and less self-referred “churning”. The central message is that policies directed at moving towards UHC need to focus particularly on alleviating the burden and barriers faced by the most disadvantaged.

An important limitation of this study is that respondents included only patients who had eventually received a diagnosis and were being treated in a hospital at the time the research was conducted. Many of those with cancer in Tanzania may not receive a diagnosis at all [12]. Our respondents’ experience does however offer some implications for policy to speed up diagnosis, in order to identify cancers at earlier stages when treatment can be more effective.

## Conclusion

Late diagnosis is a root cause, in Tanzania as in many low- and middle-income countries, of cancer treatment starting at advanced stages, undermining treatment efficacy and survival rates. While Tanzania’s policy of free public sector cancer treatment has made treatment accessible to patients on low incomes and without insurance, reaching a diagnosis is shown, for a set of 62 cancer patients, to have been slower and more expensive the greater their socio-economic disadvantage.

These findings imply that moves towards UHC, responding to the rising need for cancer care, must focus particularly on reducing costs and other barriers faced by those with low education, low incomes and vulnerable employment status. Reducing these barriers to reaching a diagnosis can reduce delays, help to down-stage the moment at which cancers begin to be treated, and create movement towards greater social justice in the design of universal health coverage.

## Supplementary Information

**Additional file 1.**

## Data Availability

The project from which these data are drawn is ongoing. Data are not yet available in appropriate form for archiving. Consistent with ESRC, UK, requirements, data from the project will be archived for use by researchers at the end of the project. Information on the archiving schedule can be provided by authors on request.

## Abbreviations

CIF: Community Health Fund
LMICs: Low- and middle-income countries
NHIF: National Health Insurance Fund
OOP: Out-of-pocket
ORCI: Ocean Road Cancer Institute
TZS: Tanzanian Shillings
USD: United States dollars
UHC: Universal Health Coverage

## Footnotes

1 Spending per head at current exchange rate; USD 112 at Purchasing Power Parity [2].

2 There was no further back-translation from English to Kiswahili to cross-check.

3 See funding statement and acknowledgements. The broader project brings together researchers from Tanzania, Kenya, India and the UK to investigate the opportunities and challenges of linking industrial development and health systems innovation in order to widen access to cancer care in Tanzania and Kenya. In Tanzania 21 health professionals were also interviewed individually, alongside focus group discussions in each hospital. These interviews are not analysed here.

4 At exchange rate TZS 2300 = 1 USD, chosen to reflect exchange rates in late 2019.

5 Cases give authors’ summaries of individual patients’ trajectories.

6 Correlation coefficient between number of moves and length of delay = 0.49.

7 Date of diagnosis of one of these patients is unclear, hence omitted from Table 4.

8 Many respondents were able to recall their payments in considerable detail; to reduce underestimates, results in Tables 6 onwards include only data from respondents able to recall 70% or more of the payments for events for which payment was required.

9 We have transport cost data only for those interviewed in the public sector.

10 A social insurance fund for health care that has expanded from public employees to cover a wide range of groups and individuals; estimated population coverage 9% in 2019 (https://www.nhif.or.tz/pages/profile#gsc.tab=0 consulted 22/07/21). NHIF offers various levels of cover for beneficiaries.

11 Figures do not add to 100 because of rounding.
