# Supplementary material for: Patients’ pathways to cancer care in Tanzania: documenting and addressing social inequalities in reaching a cancer diagnosis
Source: BMC Health Serv Res. 2022 Feb 12;22:189. doi: 10.1186/s12913-021-07438-5 (PMC8841053; doi:10.1186/s12913-021-07438-5)
Supplement: Supplementary file 1 — Additional file 1. [file 12913_2021_7438_MOESM1_ESM.pdf]

CANCER PATIENTS AND CANCER SURVIVORS QUESTIONNAIRE

COUNTYREGION/ CODE: _________/_______/

HEALTH FACILITY CODE: ______/______/

FACILITY LEVEL: _____/_______

SECTION I: Socio-demographic section

1.	**Sex**		Male		Female	

No	Questions	Categories
**2.**	**Are you on treatment?**	
		**Yes**
		**No**
**3.**	**Age (or birth date)_______**	18-24 years old
		25-34 years old
		35-44 years old
		45-54 years old
		55-64 years old
		65-74 years old
		75 years or older
		refused to answer
**4.**	**What is the highest level of school you have completed?**	**1.** Never attended school
		**2.** Nursery to class 5
		3. Class 6 to 7
		4. Form 1 to 2
		5. Form 3 to 4
		6. Form 5 to 6
		7.Trade/technical/ vocational training

		8. First Degree
		9. Higher degree
		10. Other (Specify) …………………….
**5.**	**What is your marital status?**	Single (never married)
		Married
		Cohabiting
		Widowed
		Divorced/ Separated
		Other (specify) ______________
**6.**	**How many people live with you?**	Adult
		Children
**7.**	**What is your current occupation?**	1. Student
		2. Employed/Professional (Teacher, doctor, nurse, manager, accountant etc.)
		3. Casual worker (attendant in hotel, bar, shop, house help, gardener)
		4. Housewife
		5. Business (self-employed)
		6. Farmer (large scale, subsistent farming, gardening)
		7. Unemployed
		8. Other(specify) ___________________________________
**8.**	**What was your occupation prior to the illness?**	1. Student<br>2. Employed/Professional (Teacher, doctor, nurse, manager, accountant etc.)<br>3. Casual worker (attendant in hotel, bar,

		shop, house help, gardener)<br>4. Housewife<br>5. Business (self-employed)<br>6. Farmer (large scale, subsistent farming, gardening)<br>7. Unemployed<br>8. Other(specify)
**9.**	**What is your main source of income?**	
**10.**	**Approximately how much is your household income per month?**	
**11.**	**Where is your home town/city/village?**	Write out patient' answer: village/town/city<br>___________________________________
		Don't know
		Refused to answer

SECTION II: PATIENTS CARE PATHWAYS

11. **We are now going to talk about your journey since you first felt unwell until now. Tell me when did you first feel you were unwell?**

How long ago?	What happened next?	What did you do?	Where was this?	What was done?	How much did you pay? (note down if they paid it with their own funds or received help from other people)	Did any insurance cover part of the cost? Which?	How did you travel (if in location, patient only says local healer, or local market, ask how long it took to get there)	How much did transport cost (if mode of transport prompt incurrence of costs)

Patient’s perspective on their experiences

12. **Was there anything you needed which you did not get?**

Yes ☐ **No** ☐

If yes,

What?	Not available	Too expensive	Too far	unacceptable

13. **Was there anything you decided not to do because it was unacceptable to you? Yes, no**

If yes, give details ____

(check if they consulted alternative health systems)
If they did:

14. **Why did you resort to alternative options?**
If they did not?
Did you ever consider alternative medicine options? Yes _______, No ______
If no, why not?____

15. **Given the story you have just told, what was done well?**

Early diagnosis;
Rapid diagnosis;
Short wait for test;
Short wait for surgery;
Good attitude of professionals<br>Effective treatment;
Good pain management;
Insurance coverage available;

		Other specify…………………….
16.	**Has this experience impacted on your work? [check all that apply]**<br>☐ Yes ☐ No ☐ N/A<br>_____Unable to work____.<br>_____Less time working, how much time? _____________<br>_____Changed own activity, please specify_____________.<br>_____________Changed activity by someone else, please specify	
17.	**Have you told your friends or family about your illness?**	My family
		My friends
		Both
		Neither
18.	**Who are your main sources of support?**	Community health workers
		Volunteers
		Family
		Friends
		Support groups
		Faith groups
	Other ____	
	Thank you very much. Anything else you would like to tell us<br>____	
